# EST12 regulates Myc expression and enhances anti-mycobacterial inflammatory response *via* RACK1-JNK-AP1-Myc immune pathway

**DOI:** 10.3389/fimmu.2022.943174

**Published:** 2022-08-08

**Authors:** Jian Wu, Feng-Ling Luo, Yan Xie, Huan Xiong, Yadong Gao, Guanghui Liu, Xiao-Lian Zhang

**Affiliations:** ^1^ Hubei Province Key Laboratory of Allergy and Immunology and Department of Immunology, Wuhan University School of Basic Medical Sciences, Wuhan, China; ^2^ Department of Allergy, Zhongnan Hospital of Wuhan University, Wuhan, China; ^3^ State Key Laboratory of Virology, Frontier Science Center for Immunology and Metabolism and Medical Research Institute, Wuhan University, Wuhan, China; ^4^ Wuhan Research Center for Infectious Diseases and Cancer, Chinese Academy of Medical Sciences, Wuhan, China

**Keywords:** *Mycobacterium tuberculosis (M.tb)*, Myc, Rv1579c/EST12, classical macrophage activation, IL-6, TNF-α

## Abstract

c-Myc (Myc) is a well-known transcription factor that regulates many essential cellular processes. Myc has been implicated in regulating anti-mycobacterial responses. However, its precise mechanism in modulating mycobacterial immunity remains elusive. Here, we found that a secreted Rv1579c (early secreted target with molecular weight 12 kDa, named EST12) protein, encoded by virulent *Mycobacterium tuberculosis* (*M.tb*) H37Rv region of deletion (RD)3, induces early expression and late degradation of Myc protein. Interestingly, EST12-induced Myc was further processed by K48 ubiquitin proteasome degradation in E3 ubiquitin ligase FBW7 dependent manner. EST12 protein activates JNK-AP1-Myc signaling pathway, promotes Myc binding to the promoters of IL-6, TNF-α and iNOS, then induces the expression of pro-inflammatory cytokines (IL-6 and TNF-α)/inducible nitric oxide synthase (iNOS)/nitric oxide (NO) to increase mycobacterial clearance in a RACK1 dependent manner, and these effects are impaired by both Myc and JNK inhibitors. Macrophages infected with EST12-deficiency strain (H37RvΔEST12) displayed less production of iNOS, IL-6 and TNF-α. In conclusion, EST12 regulates Myc expression and enhances anti-mycobacterial inflammatory response *via* RACK1-JNK-AP1-Myc immune pathway. Our finding provides new insights into *M.tb*-induced immunity through Myc.

## Introduction

Tuberculosis (TB) seriously threatens human health and causes a huge medical burden. According to the Global tuberculosis report 2021, it’s estimated that 9.87 million people suffered from TB, and 1.28 million TB associated deaths occurred in 2020 worldwide (1). As the only available TB vaccine, Bacille Calmette-Guérin (BCG) has limited protective efficacy in adults. Lack of effective vaccine, emergence of extensively drug-resistant tuberculosis (XDR-TB) and the side effects of toxic chemotherapeutic agents make it urgently needed to unveil the precise mechanisms that regulate mycobacterial immune response.

Sixteen regions of deletion (RDs), which are present in *M.tb* H37Rv and part of *Mycobacterium bovis*, but absent in BCG, have been identified by comparative genomic analysis (2). Recently, more and more functions of the RD proteins have been reported, suggesting that RD region-encoded proteins play important roles in the pathogenicity and immunogenicity of *M.tb*. For example, 6-kDa early secreted antigenic target (ESAT6), 10-kDa culture filtrate protein (CFP10) and MPT64, encoded by RD1-2, contribute to the survival or persistent growth of *M.tb* in macrophages (3, 4). Rv1768 of RD14 was recently identified to promote *M.tb* survival in macrophages by regulating NF-κB-TNF-α signaling and arachidonic acid metabolism through interacting with S100A9 (5). However, functions of most other RD-encoding proteins are unknown yet.

As the primary host cell for *M.tb*, macrophages eliminate intracellular *M.tb* mainly by secreting pro-inflammatory cytokines such as IL-6 and TNF-α. Macrophages also act as antigen presenting cells and present *M.tb* antigens to specific T cells through major histocompatibility complex (MHC) and activate specific CD4^+^ T and CD8^+^ T response (6). It’s well-known that pro-inflammatory M1 macrophages play a critical role in eliminating *M.tb*, while M2 macrophages are beneficial for the survival of *M.tb* and the formation of granulomas (7).

Myc is a versatile oncoprotein which regulates cell growth, differentiation, genome instability, apoptosis, etc. (8). As a prominent oncogene, the Myc target gene network is estimated to comprise about 15% of all genes from flies to humans (9) and is involved in various physiological functions and clinical diseases including TB. Mycobacteria including BCG, *M. avium*, *M. chelonae*, or *M. kansasii* have been reported to induce Myc expression (10). It is interesting that multifunctional proto-oncogene myc could be up-regulated by different species of mycobacteria, and plays a critical role in anti-mycobacterial responses (10). It was also reported that ESAT6 could downregulate lipopolysaccharide induced Myc expression *via* ERK1/2 signal pathway (11). However, the role and mechanism of Myc in modulating mycobacterial immunity during virulent *M.tb* H37Rv infection remain elusive.

Recently we have reported a secreted *M.tb* Rv1579c (early secreted target with molecular weight 12 kDa, named EST12) protein, encoded by virulent *M.tb* H37Rv RD3, binds to the receptor for activated C kinase 1 (RACK1) of macrophage and activates a RACK1–NLRP3–gasdermin D pyroptosis–IL-1β immune pathway (12). In the present study, we further found that EST12 protein induced the early expression and late K48 ubiquitination and degradation of Myc protein. EST12 activated RACK1-JNK-AP1-Myc signaling pathway which contributes to anti-mycobacterial inflammatory response of macrophages. Our study provides the first proof that Myc acts as a critical host transcriptional factor for mycobacterial EST12-induced anti-mycobacterial response and plays a pivotal role in *M.tb*-induced innate immunity.

## Materials and methods

### BMDM preparation and cell culture

Bone marrow-derived macrophages (BMDMs) were isolated from the tibia and fibula of C57BL/6 mice and induced to M0 with 50 ng/mL M-CSF (416-ML, R&D Systems, USA) for 6 days. For M1 polarized macrophages, M0 BMDMs were stimulated with 100 ng/ml LPS (L4391,Sigma, USA) and 20 ng/ml IFN-γ (485-MI, R&D Systems, USA) for 48 h. For M2 polarized macrophages, M0 BMDMs were stimulated with 20 ng/ml IL-4 (404-ML, R&D Systems, USA) and 20 ng/ml IL-13 (413-ML, R&D Systems, USA) for 48 h. Mouse RAW264.7 cells (C7505) and human THP1 cells (GDC0100) were originally obtained from China Center for Type Culture Collection (CCTCC, Wuhan, China). Both RAW264.7 and THP1 cells used were in less than 10 passage numbers (13). All above cells were cultured in Dulbecco’s Modified Eagle Medium (DMEM, Gibco) supplemented with 10% fetal bovine serum (FBS, Gibco).

### Bacterial strains

*M.tb* H37Rv [strain American Type Culture Collection (ATCC) 27294], *M.tb H37R*vΔEST12 (ΔEST12), *H37Rv*ΔEST12::EST12 (ΔEST12::EST12) and *M. bovis* BCG (Pasteur strain ATCC 35734) were maintained in our lab according to previous publications (12, 14, 15). Briefly, the mycobacterial strains were cultured in Middlebrook 7H9 broth (7H9) supplemented with 10% oleic acid–albumin–dextrose–catalase (OADC) and 0.05% Tween 80 (Sigma-Aldrich) or on Middlebrook 7H10 agar (BD Biosciences). *Escherichia coli* (*E. coli*) DH5α (strain ATCC 25922) and *E. coli* BL-21 (stain ATCC BAA-1025) were propagated from laboratory stocks (School of Medicine, Wuhan University, Wuhan, China), and cultured in LB medium.

### Animals

WT C57BL/6 mice (6–8-week-old) were purchased from the Centre of Animal Experiments of Wuhan University. RACK1 knock out (KO) (12), IL-6 KO, TLR2 KO and TLR4 KO (5) C57BL/6 mice were maintained in Animal Laboratory Center of Wuhan University. All animals received humane care according to the criteria outlined in the Guide for the Care and Use of Laboratory Animals prepared by the National Academy of Sciences and published by the National Institutes of Health (NIH publication no. 86-23, revised 1985). All animal protocols were approved by the Institutional Animal Care and Use Committee of the Institute of Model Animals of Wuhan University (nos. S01319070R and S01317012S).

### Recombinant EST12 protein preparation

*E. coli* BL21 (DE3) were transformed with expression plasmid pET-28a-EST12 with 6×His-tag. Expression of recombinant EST12 protein was induced by isopropyl-β-D-thiogalactopyranoside (IPTG) at 25°C for 16 h. The bacteria were resuspended in phosphate-buffered saline (PBS) and disrupted by dynamic high-pressure homogenization, then the cell debris was removed by centrifugation at 14,000 g at 4°C for 15 min. The supernatants were incubated with Ni-agarose pre-equilibrated with 10 mM imidazole solution [25 mM Tris-HCl, 200 mM NaCl, and 10 mM imidazole (pH 7.4)]. The recombinant proteins were eluted with 20, 40, 60-, 80-, 100- and 200-mM imidazole buffer (with 25 mM Tris-HCl and 200 mM NaCl, pH 7.4). The purified recombinant proteins were further purified using endotoxin-free purification polymyxin B columns and analyzed by sodium dodecyl sulfate-polyacrylamide gel electrophoresis (SDS-PAGE) and Western blot with antibody against His-tag. EST12 protein concentration was determined by bicinchoninic acid (BCA) method.

### Transcriptional sequencing

RAW264.7 cells (1 × 10^7^) were stimulated with 2 μM EST12 protein for 3 h. The total RNA of treated cells was extracted with Trizol and mRNA sequencing was performed by Allwegene (Allwegene Biotech, www.allwegene.com).

### Reverse transcription-quantitative real time PCR

The total RNA from cells was extracted with Trizol reagent (Invitrogen Corp, Carlsbad, CA, USA) and reverse-transcribed using a cDNA reverse transcription kit (Toyobo, Osaka, Japan) with Oligo dT primers. The reverse transcribed cDNA was used as a template for qPCR reactions along with SYBR Green Real time PCR Master Mix (Toyobo, Osaka, Japan) and 0.4 μM primers (primer sequences shown in **Supplementary Table 1**, primers were synthesized by TSINGKE, Wuhan). The qPCR reactions were run on an ABI Step One Plus (Applied Biosystems) using standard cycling conditions. Relative RNA levels were calculated by the comparative cycle threshold (CT) method (2^−ΔΔCT^ method) (16), where CT represents the amplification cycle number at which the fluorescence generated within a reaction rises above a defined threshold fluorescence and ΔΔCT = experimental groups (Ct_Target gene_ − Ct_GAPDH_) − control groups (Ct_Target gene_ − Ct_GAPDH_). The mRNA levels of each gene in the experimental groups were then presented as the fold levels relative to the blank control groups calculated by the following formula: 2^−ΔΔCT^.

### Cell transfection

RAW264.7 cells were cultured in DMEM supplemented with 10% (v/v) heat-inactivated FBS. After reaching 70%-90% confluence, transient transfection was performed with jetPEI-Macrophage (Polyplus) following the manufacturer’s instructions. For siRNA transfection, the silencing RNA (siRNA) of E3 ubiquitin ligase FBW7 and control siRNA were synthesized from TSINGKE company (Wuhan, China). RAW264.7 cells were transfected with 100 nM siRNA. Transfection efficiency was measured by Western blot after 48 h post transfection. The sequences of siRNAs were listed in **Supplementary Table 2**.

### Nuclear/cytoplasmic fractionation and Western blot analysis

Cells were harvested and lysed in RIPA buffer supplemented with protease inhibitors (P-8340, Sigma-Aldrich, US). For cell localization analysis of target proteins, nuclear and cytoplasmic proteins of EST12-stimulated RAW264.7 cells were extracted with Nuclear and Cytoplasmic Protein Extraction Kit (P0027, Beyotime, China) according to the manufacturer’s recommended protocol. Protein concentrations in cell lysates or extracted nuclear and cytoplasmic proteins were determined with the Pierce™ BCA Protein Assay Kit (23225, Thermo Fischer Scientific, US), and 60 μg of each protein sample was loaded and separated on SDS-PAGE (5% for concentration gel, 12% for separation gel) after being denatured at 98°C for 8 min with SDS loading buffer. Proteins were transferred onto a nitrocellulose membrane which was subsequently blocked for 2 h with a 5% BSA solution (prepared in TBS with 0.05% Tween-20) and incubated overnight with primary antibodies (diluted at 1:1000). Myc (10828-1-AP, Proteintech, China), GAPDH (60004-1-Ig, Proteintech, China), β-Tubulin (A12289, ABclonal, China), p-JNK (AP0276, ABclonal, China), p-cJun (AP0105, ABclonal, China), p-cFos (AP0038, ABclonal, China), p-Myc S62 (AP0989, ABclonal, China), p-Myc T58 (AP0990, ABclonal, China), H3 (17168-1-AP, Proteintech, China), RACK1 (ab129084, abcam, UK), inducible nitric oxide synthase (iNOS) (A3774, ABclonal, China), Arg1 (A4923, ABclonal, China), HA-Tag (66006-2-Ig, Proteintech, China), His Tag (AE003, ABclonal, China), FBW7 (28424-1-AP, Proteintech, China), TLR2 (A19125, ABclonal, China), TLR4 (A5258, ABclonal, China). Membranes were then incubated with horse radish peroxidase (HRP)-conjugated secondary antibodies (1:5000 dilution, goat anti mouse IgG KR0026, goat anti Rabbit IgG KR0023, KeRui, Wuhan) and detected by LumiGlo Reserve™ Chemiluminescent Substrate Kit (54-61-01, Sera care, Life Sciences, MA, US). Protein bands were visualized using the UVP BioSpectrum™ 500 Imaging System with the VisionWorks^®^ images acquisition and analysis software (Analytik Jena, CA, US).

### Dual Luciferase Reporter Assay

The Dual-Luciferase^®^ Reporter Assay System (Promega, Madison, United States) was used to detect EST12-induced IL-6 and TNF-α activation on a GloMax^®^ 20/20 tube luminometer (Promega, Madison, United States). Briefly, RAW264.7 cells were co-transfected with pGL3- IL-6-luc and internal control Renilla plasmid or pGL3- TNF-α-luc and internal control Renilla plasmid. Then, 24 h post-transfection, cells were treated with 2 μM EST12 in the presence of 20 μM Myc inhibitor (10058-F4, Selleck) or not. After 1 h, the cells were harvested. Luciferase activity was measured using the Dual-Luciferase Reporter Assay System according to the manufacturer’s instructions (Promega, Madison, United States). Data were normalized for transfection efficiency by dividing firefly luciferase activity with that of Renilla luciferase.

### Cleavage under targets and tagmentation sequencing

RAW264.7 cells (1×10^7^) were stimulated with 2 μM EST12 for 1 h, then treated or untreated RAW264.7 cells were collected and sent to DIATRE Biotechnology (Shanghai, China) in dry ice for CUT&Tag sequencing. Briefly, in this method, a highly active Tn5 transposase is fused to Protein A, and a library-building linker primer is loaded to form a pA-Tn5 transposition complex. Under the guidance of Myc antibody (10828-1-AP, Proteintech, China), the pA-Tn5 transposition complex can target and cut the DNA sequence near Myc-bound region.

### Chromatin immunoprecipitation and quantitative polymerase chain reaction

2 μM EST12-stimulated RAW264.7 cells were lysed and sonicated with Bioruptor Non-Contact Automatic Ultrasonic Disruptor (Diagenode, Belgium), ultrasonic condition is 30 s on, 30 s off, 20 cycles. Then samples were incubated with beads conjugated with anti-Myc (10828-1-AP, Proteintech, China) or H3-K4 (A2357, ABclonal, China) antibody. Captured DNA was purified with DNA Purification Kit (DP214, TIANGEN, Beijing) and used for quantitative PCR analysis. The primers were synthesized from TSINGKE (Wuhan, China) and listed in **Supplementary Table 3**.

### Electrophoretic mobility shift assay

RAW264.7 cells (1×10^7^) were seeded into 10 cm dishes and stimulated with 2 μM EST12 for 1 h, the nuclear extracts were prepared by Nuclear and Cytoplasmic Protein Extraction Kit (P0027, Beyotime, China). EMSA assays were done with Chemiluminescent EMSA kits (GS009, Beyotime, China). 5 μg nuclear extracts were incubated with 1× binding buffer and biotin-labeled probe or unlabeled probes for 30 min at room temperature (RT). The samples were electrophoresed on a 6% polyacrylamide gel in 0.5 × TBE at 100 V for 1 h and transferred onto a nylon membrane in 0.5 × TBE at 300 mA for 1 h. After transfer and 254 nm UV cross-linking, the membrane was detected with Streptavidin-HRP. Biotin-labeled and unlabeled probes were synthesized from TSINGKE (Wuhan, China) and listed in **Supplementary Table 4**.

### Plasmid DNA construction

cDNA of Myc was cloned into pcDNA3.1 vector (Invitrogen, Carlsbad, CA) using the templates kindly provided by Professor Jiahuai Han of Xiamen University, and the restriction enzyme cutting sites are *Eco*RI and *Bam*HI. The promoter region of IL-6 (1201 bp) and TNF-α (1238 bp) were cloned into pGL3-basic by method of homologous recombination, the genome of RAW264.7 cells was extracted with TIANTGEN Cell Genomic DNA Extraction Kit (TIANGEN, DP304, China) and used as template. Restriction enzymes *Xho*I and *Hin*dIII were used to digest the pGL3-basic. Primers were synthesized from TSINGKE (Wuhan, China) and listed in **Supplementary Table 5**.

### Enzyme-linked immunosorbent assay and NO measurement

Cytokine production of treated cells was measured by ELISA using the corresponding ELISA kits according to the manufacturers’ protocols. IL-6 (DKW1210602, Dakewe Biotech, China); TNF-α (DKW1217202 Dakewe Biotech, China); IL-10 (CME0016, 4A Biotech, China); TGF-β (DKW1217102 Dakewe Biotech, China). For NO measurement, culture supernatants were harvested after stimulation of macrophages with EST12 protein for indicated time. The release NO level was determined using the NO assay kit (Beyotime, Shanghai, China). Supernatants were added to an equal volume of Griess reagent in duplicate on a 96-well plate and incubated at room temperature for 15 min. Absorbance (540 nm) was measured and NO concentrations were estimated using a standard NO curve. The experiments were performed in triplicate.

### Immunoprecipitation

MG-132 (5 μM) pretreated RAW264.7 cells (1×10^7^) were stimulated with 2 μM EST12 protein for 0, 1 or 12 h, and then cells were collected and lysed with 700 μL RIPA buffer on ice for 20 min. After centrifuge at 14,000 g for 20 min at 4°C, 3 μg anti-Myc antibody (10828-1-AP, Proteintech, China) and 20 μL 50% protein A/G beads buffer were added and incubated at 4°C overnight, then protein A/G captured complex was eluted. Myc and FBW7 protein levels were detected by Western blot using anti-Myc antibody and anti-FBW7 antibody (28424-1-AP, Proteintech, China).

### Colony forming unit assay

For intracellular mycobacterial survival assay, RAW264.7 cells (5 ×10^5^) were infected with WT (H37Rv), EST12 deficiency H37Rv (H37RvΔEST12) or complement strain (H37RvΔEST12::EST12) at MOI=10 for 4 h. RAW264.7 cells were then washed with PBS for three times to remove extracellular bacteria, and DMEM medium supplemented with gentamicin (10 μg/ml) was added and further incubated in the presence or absence of Myc inhibitor (20 μM) (10058-F4, MCE, shanghai) or JNK inhibitor (10 μM) (JNK-IN-7, Topscience, shanghai) for 4 h, 8 h, 24 h and 48 h, respectively. Cells were lysed and intracellular bacteria were plated and counted on 7H10 medium.

For BCG infection assay, WT BMDMs (5 ×10^5^) or IL-6 KO BMDMs were infected with BCG at MOI=10 for 4 h, then cells were washed with PBS for three times to remove extracellular bacteria, and then purified EST12 (2 μM) was added with or without the presence of TNF-α neutralizing antibody (MAB4101, R&D Systems, USA) (10 μg/ml) and further incubated for 8 h. Cells were lysed and intracellular bacteria were plated and counted on 7H10 medium.

### Immunofluorescence microscopy

RAW264.7 cells were pretreated with MG-132 (5 μM) for 1 h and treated with 2 μM purified EST12 protein for indicated time. Media was removed from stimulated macrophages and the dishes were submerged in 4% paraformaldehyde (PFA). The fixed dishes were submerged in PBS to remove residual PFA and then cells were permeabilized with 0.1% Triton X-100 in PBS for 5 minutes at room temperature (RT), washed in PBS and blocked in PBS containing 10% donkey serum. Primary antibodies against FBW7 (28424-1-AP, Proteintech, China) and Myc (M4439, Sigma-Aldrich, USA) were used at a 1:50 dilution in PBS with 3% serum and incubated at 4°C overnight. Then Alexa Fluor 594-conjugated goat anti-rabbit IgG (A-11037, ThermoFisher, USA) or Alexa Fluor 488-conjugated goat anti-mouse IgG (A28175, ThermoFisher, USA) was used at 1:500 dilution in PBS with 3% FBS and incubated at RT for 1 h. Dishes were washed to remove the unbound secondary antibody and the cellular nuclei DNA was stained with DAPI. Fluormount-G Anti-Fade Mounting Medium (Southern Biotech) was added to each well to protect the fluorescent signal. Confocal fluorescence microscopy was performed using ZEISS LSM880 microscope and representative images were captured.

### Flow cytometry

BMDMs (1×10^6^) were stimulated with 2 μM EST12 protein for 12 h or 24 h, then cells were collected and incubated with APC-F4/80 (QA17A29, BioLegend, USA), FITC-CD80 (16-10A1, BioLegend, USA) and PE-CD86 (A17199A, BioLegend, USA) at 4°C for 30 min. Then cells were washed three times with PBS and subsequently used for flow cytometry using FACSCalibur (BD Biosciences) flow cytometer and analyzed by FlowJo 10.7.

### Statistical analysis

Results were plotted by GraphPad Prism v9 (GraphPad Software, San Diego, CA, USA). For analysis of the statistical significance of differences between two groups, two-tailed unpaired Student’s t-tests was used. For analysis of the statistical significance of differences among more than two groups, one-way ANOVA with Tukey’s multiple comparisons test was used. To assess the statistical significance of differences among multiple groups when the experimental design involved multiple conditions, such as time points or bacterial types in addition to differences in inhibitor treatment, two-way ANOVA with Tukey’s multiple comparisons test was used. Data are presented as mean ± SEM, n=3. P values < 0.05 were considered statistically significant. *, *p* < 0.05; **, *p* < 0.01; ***, *p* < 0.001.

## Results

### Mycobacterial EST12 induces early Myc expression in macrophages during *M.tb* infection

**Figure 1:**
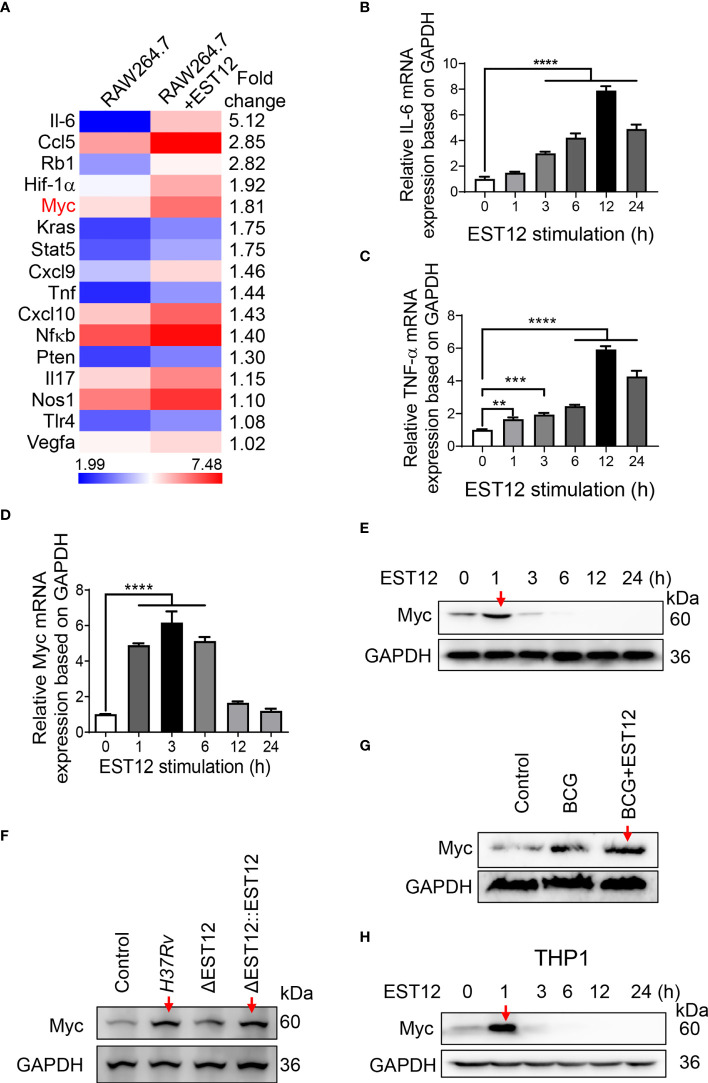
Mycobacterial EST12 induces early Myc expression in macrophages during *M.tb* infection. **(A)** RAW264.7 cells were stimulated with EST12 protein (2 μM) for 3 h, then the differentially expressed genes were detected by transcriptional sequencing. The inflammation and cancer-related genes were displayed in heatmap.**(B-E)** RAW264.7 cells were stimulated with EST12 protein (2 μM) for the indicated time. The mRNA levels of IL-6 **(B)**, TNF-α **(C)** and Myc **(D)** were determined by RT-qPCR, and the protein level of Myc was determined by Western blot **(E)**. **(F)** RAW264.7 cells infected with WT *M.tb* H37Rv, H37RvΔEST12 or H37RvΔEST12::EST12 for 24 h, and Myc expression in each cell lysates were detected by Western blot. **(G)** RAW264.7 cells were infected with BCG (MOI=10) for 24 h, and then stimulated with or without EST12 protein (2 μM) for 1 h, the expression of Myc was detected by Western blot. **(H)** THP1 cells were induced with PMA (100 ng/ml) for 24 h and then stimulated with EST12 protein (2 μM) for the indicated time, the protein levels of Myc expression were determined by Western blot. One-way ANOVA with Tukey’s multiple comparisons test was used to assess the statistical significance in **(B–D)** (vs. 0 h). The data are expressed as the mean ± SEM of three independent experiments, *p* > 0.05, not significant (ns), **p < 0.01, ***p < 0.001, or ****p < 0.0001.

*M.tb*-infected lungs are often accompanied by chronic inflammation and it has been reported that chronic TB infection can induce lung squamous cell carcinoma in a mouse model (17), therefore, we examined the inflammation- and cancer-related differentially expressed genes in EST12**-**stimulated mouse RAW264.7 cells through transcriptome sequencing. Recombinant EST12 protein was purified and used to treat RAW264.7 cells for 3 h (**Figure S1A**; **Figure 1A**). As shown in **Figure 1A**, EST12 not only significantly increased the expression of pro-inflammatory cytokine genes (such as IL-6 and TNF-α) (**Figure 1A**), but also induced the expression of four oncogenes (RB1, Myc, KRAS and PTEN). We confirmed that EST12 significantly upregulated the expression of IL-6 and TNF-α by RT-qPCR (**Figures 1B, C**). Furthermore, we found that Myc was the most significantly upregulated by EST12 protein treatment (**Figure 1D**), although EST12 also induced early expression of RB1, KRAS and PTEN oncogenes at mRNA level (**Figures S1B–D**). Next, the Myc protein level in EST12-stimulated RAW264.7 cells was analyzed by Western blot (**Figure 1E**). As shown in **Figures 1D, E**, EST12 upregulated the early transcription of Myc in RAW264.7 cells (within 6 h stimulation, **Figure 1D**) and transitorily activated Myc protein expression with decreased Myc protein levels at later stage (1 h post-stimulation of EST12) (**Figure 1E**). Furthermore, EST12 KO strain (H37RvΔEST12) infection decreased the expression of Myc protein in RAW264.7 cells compared with WT H37Rv and the complementary strain H37RvΔEST12::EST12 (H37RvΔEST12 strain rescued with EST12) infection (**Figures 1F**; **S1E, F**). We also found that BCG infection also induced Myc expression in RAW264.7 cells (**Figure 1G**), suggesting that other factors of mycobacterium, except EST12, might also be involved in induction of Myc expression. Consistently, exogenous EST12 protein could also upregulate Myc expression during BCG infection in RAW264.7 cells (**Figure 1G**). Similarly, we found that EST12 significantly induced the expression of Myc in human-derived macrophage THP1 cells (**Figure 1H**). Altogether, we found that mycobacterial EST12 could induce early expression of Myc protein in macrophages.

### EST12 induces the expression and phosphorylation of Myc through JNK-AP1-Myc signaling pathway

**Figure 2:**
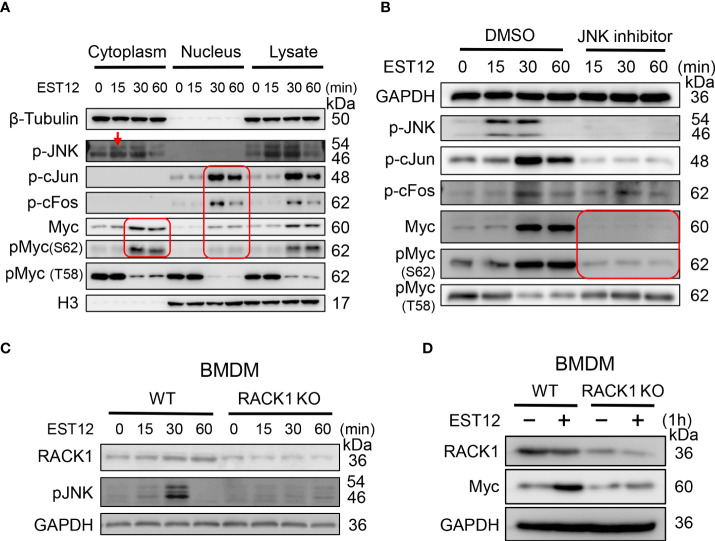
EST12 induces the expression and phosphorylation of Myc through JNK-AP1-Myc signaling pathway. **(A)** RAW264.7 cells were treated with EST12 protein (2 μM) for the indicated time, then the nucleus and cytoplasm were separated. The expression of p-JNK, p-c-Jun, p-cFos, Myc and p-Myc (S62/T58) in the cytoplasm, nucleus and total cell lysate were detected by Western blot. **(B)** RAW264.7 cells were pretreated with JNK inhibitor (10 μM) for 1 h and then stimulated with EST12 for the indicated time, the expression of p-JNK, p-AP1, Myc and p-Myc (S62/T58) was analyzed by Western blot. **(C, D)** WT or RACK1 KO BMDMS were stimulated with EST12 protein (2 μM) for the indicated time, the expression of p-JNK **(C)** and Myc **(D)** was detected by Western blot.

To further unveil the molecular pathways involved in EST12-induced Myc activation, we extracted the nuclear and cytoplasmic proteins from EST12-treated RAW264.7 cells. Time-course and Western blot analysis showed that EST12 induced and activated cytoplasmic JNK phosphorylation (p-JNK) (reached peak at 15-30 min), nuclear p-cJun and p-cFos (p-AP1) (AP1 consists of c-Jun and c-Fos) (reached peak at 30 min) and both cytoplasmic and nuclear p-Myc at Ser62 (reached peak at 30 min~60 min) (**Figure 2A**), while p-Myc (Thr58) expression was not activated, but displayed a decrease within 1 h stimulation of EST12 (**Figure 2A**). These results suggest that EST12 activates early phosphorylation of Myc at Ser62 and p-JNK-p-AP1-p-Myc (S62) signaling. Further, JNK inhibitor significantly blocked EST12-induced p-JNK, p-p-cJun, p-cFos and p-Myc (S62) expression (**Figure 2B**).

Our previous study has demonstrated that *M.tb* EST12 protein binds RACK1 and activates a RACK1–NLRP3–gasdermin D pyroptosis–IL-1β immune pathway (12). Here we further demonstrated that EST12 induced p-JNK (**Figure 2C**) and Myc (**Figure 2D**) expression in WT macrophages but not in RACK1 KO macrophages, suggesting that EST12 induced p-JNK and Myc expression dependent on RACK1. Therefore, these data suggest that EST12-RACK1 interaction induces the expression and phosphorylation of Myc through JNK-AP1-Myc signal pathway.

### *M.tb*-EST12 induces the production of proinflammatory cytokines and iNOS/NO at later stage to increase mycobacterial clearance

**Figure 3:**
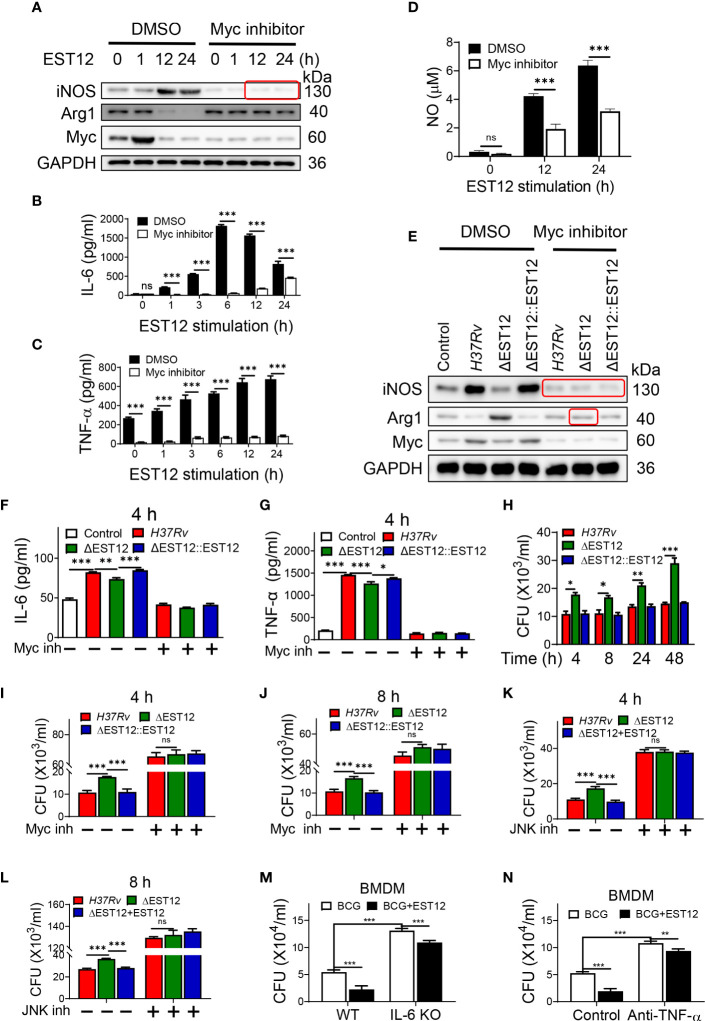
EST12-induced Myc-mediated classical M1 cytokines expression and activation eliminates intracellular *M.tb*. **(A–D)** RAW264.7 cells were pretreated with Myc inhibitor (10058-F4, 20 μM) (Myc inh) for 1h, and then stimulated with EST12 protein for the indicated time, the expression of iNOS and Arg1 was determined by Western blot **(A)**; the levels of the secreted IL-6 **(B)** and TNF-α **(C)** were determined by ELISA; NO levels in the supernatant were determined as described in Materials and Methods **(D)**. **(E–G)** RAW264.7 cells were infected with each indicated *M.tb* strains at MOI=10 and cultured with or without Myc inhibitor for 24 h, the expression of iNOS and Arg1 was determined by Western blot **(E)**; the levels of the secreted IL-6 **(F)** and TNF-α **(G)** were determined by ELISA. **(H–L)** RAW264.7 cells were infected with indicated *M.tb* strains and cultured with or without Myc inhibitor or JNK inhibitor. The cells were lysed and the CFUs of the intracellular *M.tb* were enumerated at 4 h, 8 h, 24 h or 48 h post infection. **(M)** BMDMs isolated from WT or IL-6 KO C57BL/6 mice were infected with BCG at MOI=10. Then BMDMs were stimulated with EST12 protein (2 μM) for 8 h. The cells were lysed and the CFUs of intracellular *M.tb* were enumerated. **(N)** BMDMs from WT C57BL/6 mice were infected with BCG at MOI=10. Then, BMDMs were stimulated with EST12 (2 μM) with or without TNF-α neutralizing antibody (10 μg/ml) for 8 h. The cells were lysed and the CFUs of the intracellular *M.tb* were enumerated. One-way ANOVA with Tukey’s multiple comparisons test was used to assess the statistical difference in **(F, G)**. Two-way ANOVA with Tukey’s multiple comparisons test was used to assess the statistical significance in **(B–D)** and **(H–N)**. The data are expressed as the mean ± SEM of three independent experiments. *p* > 0.05, not significant (ns), *p < 0.05, **p< 0.01, or ***p < 0.001.

Proinflammatory cytokines IL-6 and TNF-α could increase the elimination of intracellular *M.tb* (10). As EST12 increased the expression of macrophage IL-6 and TNF-α (**Figure 1A**), we wonder whether EST12 could alter the characteristics of macrophage activation and polarization through activating Myc. Furthermore, we pretreated RAW264.7 cells with DMSO or Myc inhibitor 10058-F4, followed by stimulation with EST12 protein. Western blot analysis showed that EST12 significantly induced early Myc expression (1 h post-stimulation of *M.tb*-EST12) (lane 2 vs. lane 1, **Figure 3A**). At later stage, 12-24 h post-stimulation of EST12 increased classical M1 polarization-related factor iNOS expression (lane 3 and 4, **Figure 3A**) but inhibited M2 polarization factor Arg1 or Myc expression (Line 3 and 4, **Figure 3A**). EST12 regulated Myc expression, in a trend consistent with that of Arg-1 expression which also showed decreased expression after 12 h stimulation of EST12 and early expression at 0-1 h post-stimulation of EST12 (**Figure 3A**). Myc inhibitor treatment blocked EST12-induced iNOS expression (**Figure 3A**).

Consistently, ELISA analysis showed that EST12 significantly induced the expression of IL-6 (**Figure 3B**), TNF-α (**Figure 3C**) and NO (**Figure 3D**), but Myc inhibitor treatment blocked these effects (**Figures 3B–D**). Furthermore, Myc inhibitor blocked H37Rv infection-induced expression of iNOS and Arg-1 (**Figure 3E**), IL-6 (**Figure 3F**) and TNF-α (**Figure 3G**). However, EST12 stimulation had no effects on the production of the secreted IL-10 (**Figure S2A**) and TGF-β (**Figure S2B**) in RAW264.7 cells. We also determined that EST12 significantly upregulated the expression of CD80, CD86 of macrophages by FCM analysis (**Figures S2C, D**), and increased the production of the secreted IL-1β (**Figure S2E**) and IL-12 (**Figure S2F**), but not IFN-γ (**Figure S2G**) by ELISA.

As predicted, *M.tb* H37Rv and H37RvΔEST12::EST12 infection of RAW264.7 led to less CFUs compared to H37RvΔEST12 at 4 h, 8 h, 24 h and 48 h post-infection (**Figure 3H**). While Myc inhibitor pretreatment increased the survival of intracellular *M.tb* H37Rv and H37RvΔEST12::EST12 strains in RAW264.7 cells, and H37RvΔEST12 as well (**Figures 3I, J**), suggesting that other factors, except EST12, might also induce Myc expression. Consistently, we observed similar results in JNK inhibitor pretreated RAW264.7 cells (**Figures 3K, L**). Furthermore, exogenous EST12 protein treatment after BCG infection of macrophages could also repress intracellular BCG survival (**Figures 3M, N**). However, intracellular BCG survival was increased in IL-6 KO BMDMs (**Figures 3M**, **S3A**) or in the presence of TNF-α neutralization antibody (**Figures 3N**, **S3B**). We also observed that exogenous EST12 protein treatment could repress intracellular BCG survival in IL-6 KO BMDMs (**Figure 3M**) or in the presence of TNF-α neutralization antibody (**Figure 3N**), as well.

All these results suggest that EST12 induces early Myc expression. At later stage (12-24 h post-stimulation of *M.tb*-EST12), EST12 increased iNOS/NO expression, but decreased Arg1 or Myc expression with associated induction of IL-6, TNF-α and IL-1β to increase mycobacterial clearance. EST12 stimulation also induced the expression of CD80 and CD86, but not IL-10 and TGF-β.

### EST12 promotes Myc to bind to the promoters of IL-6 and TNF-α, and then induces IL-6 and TNF-α expression

**Figure 4:**
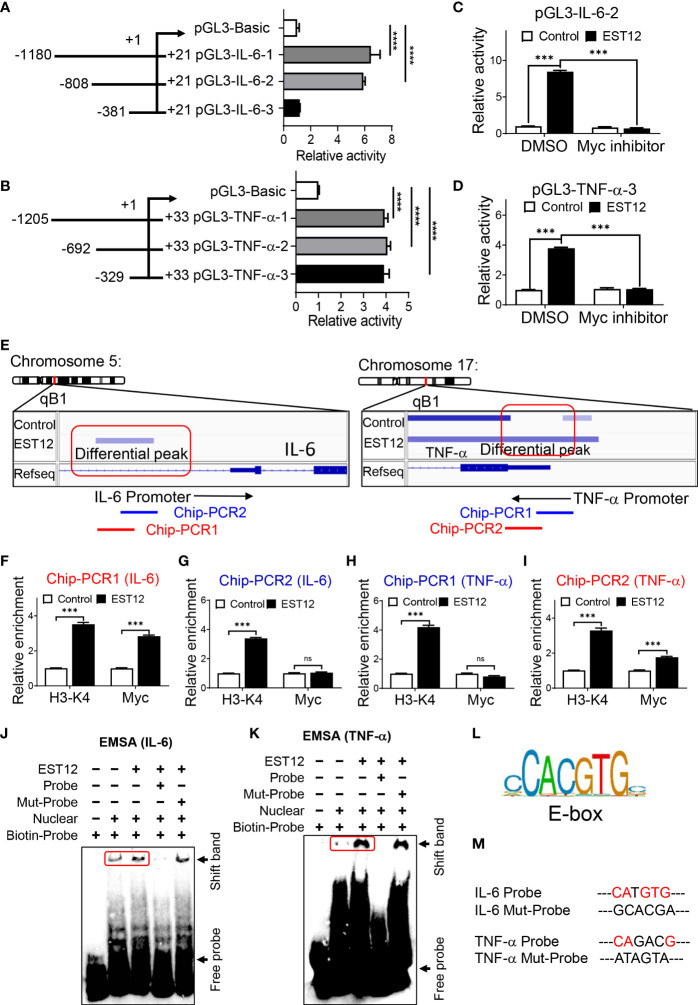
EST12 promotes Myc to bind to the promoters of IL-6 and TNF-α, and then induces IL-6 and TNF-α expression. **(A, B)** The different truncation regions of IL-6 and TNF-α promoters were cloned into pGL3-basic plasmid. Each plasmid was co-transfected with control fluorescent plasmid Renilla into RAW264.7 cells for 24 h. Then cells were stimulated with EST12 protein (2 μM) for 1 h. The cells were lysed and luciferase activities of each group were measured as described in Materials and Methods. **(C, D)** RAW264.7 cells were transfected with pGL3-IL-6-2 **(C)** or pGL3-TNF-α-3 **(D)**. Then cells were treated with Myc inhibitor (10058-F4, 20 μM) for 1 h, then stimulated with EST12 protein (2 μM) for 1 h. Then cells were lysed and the luciferase activities were measured. **(E)** RAW264.7 cells were stimulated with EST12 protein (2 μM) for 1 h, and then proceeded CUT&Tag sequencing analysis as described in Materials and Methods. **(F–K)** The effects of EST12-induced Myc binding to the promoters of IL-6 and TNF-α were analyzed by ChIP-qPCR **(F–I)** and EMSA **(J, K)**, respectively. IL-6: PCR1 (-516, -367), PCR2 (-385, -277); TNF-α: PCR1 (-223, -88), PCR2 (-106, +33). **(L)** The classical E-box motif. **(M)** Mutation sites used in **(J, K)** One-way ANOVA with Tukey’s multiple comparisons test was used to assess the statistical difference in **(A, B)**. Two-way ANOVA with Tukey’s multiple comparisons test was used to assess the statistical difference in **(C**, **D)** and **(F–I)** (vs. Control). The data are expressed as the mean ± SEM of three independent experiments. *p* > 0.05, not significant (ns), ***p < 0.001, or ****p < 0.0001.

In order to further determine whether Myc acts as a critical transcription factor for *M.tb*-EST12-induced production of IL-6 and TNF-α. We cloned the full length and different truncated sequences of IL-6 and TNF-α promoters into the pGL3 vector as shown in **Figures 4A, B** respectively. Then we performed dual luciferase assays to confirm the induction of IL-6 and TNF-α expression by EST12 protein. Our results showed that EST12 significantly upregulated the transcription of IL-6 (**Figure 4A**) and TNF-α (**Figure 4B**), and the critical regulatory sites are -808 bp to -381 bp and -329 bp to +33 bp of IL-6 and TNF-α promoter regions, respectively (**Figures 4A, B**). Furthermore, we transfected pGL3-IL-6-2 (IL-6 promoter region from -808 bp to +21 bp) and pGL3-TNF-α-3 (TNF-α promoter region from -329 bp to +33bp) into RAW264.7 cells and confirmed that EST12 stimulation significantly induced IL-6 and TNF-α activation, however, Myc inhibitor significantly blocked EST12’s inductive effects (**Figures 4C, D**). To confirm Myc binding to the promoter sequences of IL-6 and TNF-α, we analyzed the results of CUT&Tag sequencing which captured differential sequences between EST12-treated RAW264.7 group and control group with anti-Myc antibody. Our CUT&Tag results showed that EST12 induced specific differential peaks at the promoter regions of IL-6, TNF-α (**Figure 4E**) and iNOS (**Figure S3C**). We focused the further research on exploring how EST12 induces the expression of IL-6 and TNF-α through Myc. Then ChIP-qPCR assays confirmed that the critical binding site of Myc is located at the -516 bp to -367 bp, but not at -385 bp to -277 bp of IL-6 promoter region during EST12 stimulation (**Figures 4E**, left, **4F, G**). Similarly, we also found that the Myc critical binding site is located at the -106 bp to +33 bp, but not at -223 bp to -88 bp of TNF-α promoter region during EST12 stimulation (**Figures 4E**, right **4H, I**). H3-K4 was used as a positive transcriptional factor control (**Figures 4F–I**).

Next, we further performed EMSA to determine whether EST12 induced-Myc bound the IL-6 and TNF-α promoters using the corresponding 31-nucleotide DNA probe (**Supplementary Table 4**) containing the potential Myc transcription factor binding region. Nuclear protein extracts from EST12-pretreated RAW264.7 cells displayed stronger binding to the probe (**Figures 4J, K** Lane 3 vs. Lane 2) than those from untreated RAW264.7 cells. The binding was abrogated when the extract was incubated with a 10-fold excess of the unlabeled ‘competitor’ probe (**Figures 4J, K** Lane4), but when we mutated the E-box (the classical Myc binding motif) (**Figure 4L**) like sequence “CATGTG” of IL-6 promoter or “CAGACG” of TNF-α promoter (**Figure 4M**, **Supplementary Table 4**, underlined), the mutant (Mut) ‘competitor’ probe did not abolish binding, providing additional evidence for its specificity (**Figures 4J, K** Lane5). These results suggested that EST12 induced Myc binding to the promoters of IL-6 and TNF-α at E-box like sequence, and thus increased the expression of these cytokines.

### EST12 decreased Arg1 or Myc expression and increased classical M1 activation and pro-inflammatory response relying on RACK1 and JNK at later stage

**Figure 5:**
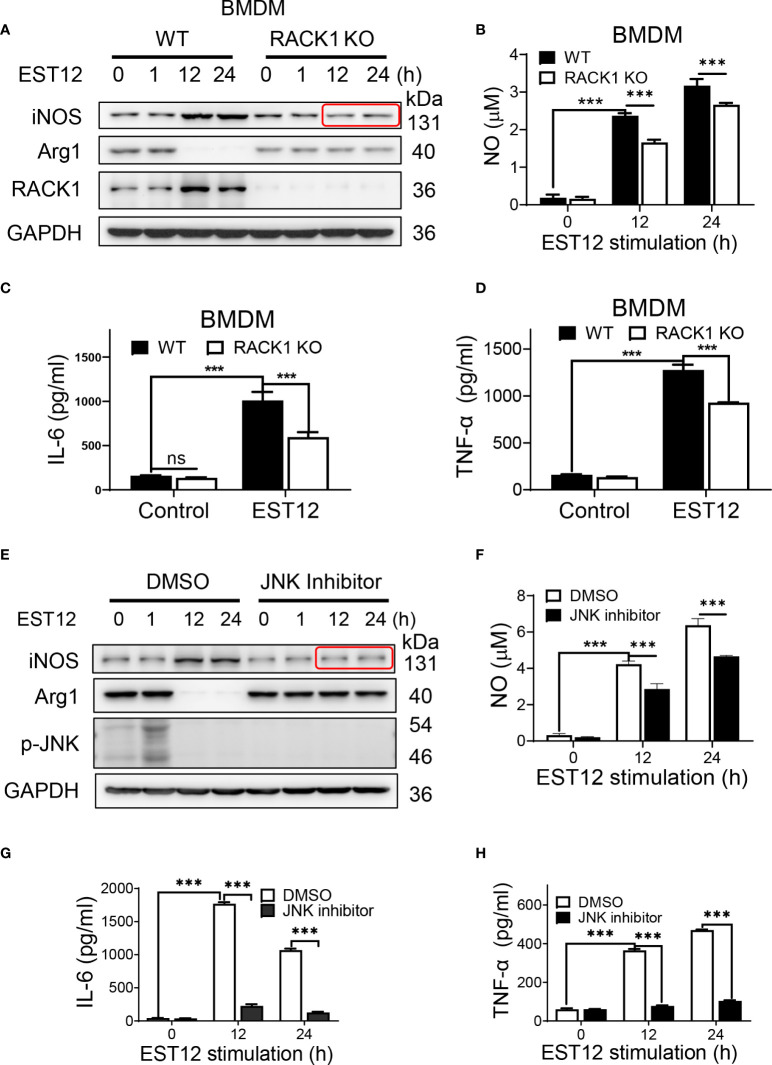
EST12 induces classical M1 activation relying on RACK1-JNK signaling pathway. **(A-D)** BMDMS isolated from WT, RACK1 KO C57BL/6 mice, respectively, were stimulated with EST12 protein (2 μM) for the indicated time, the expression of iNOS and Arg1 was determined by Western blot **(A)**; NO in the supernatant was determined as described above **(B)**; the secreted IL-6 **(C)** and TNF-α **(D)** levels were determined by ELISA. **(E-H)** RAW264.7 cells were pretreated with JNK inhibitor (10 μM), and then stimulated with EST12 protein (2 μM) for the indicated time, the expression of iNOS and Arg1 was determined by Western blot **(E)**; NO in the supernatant was determined as described above **(F)**; the expression of IL-6 **(G)** and TNF-α **(H)** were determined by ELISA. Two-way ANOVA with Tukey’s multiple comparison test was used for **(B**-**D)** and **(F**-**H)**. The data are expressed as the mean ± SEM of three independent experiments. *p* > 0.05, not significant (ns), ***p < 0.001.

As the above results showed that EST12 induced early Myc expression (1 h post-stimulation of *M.tb*-EST12), but decreased M2 polarization-related factor Arg1 or Myc expression and increased classical M1 polarization-related factor iNOS and pro-inflammatory cytokines (IL-6 and TNF-α) expression at later stage (12-24 h post-stimulation of *M.tb*-EST12, **Figure 3A**). We next examined whether these effects of EST12 relying on RACK1 and JNK. Compared with WT BMDMs, RACK1 deficiency (RACK1 KO) significantly decreased EST12-induced iNOS (**Figure 5A**), NO (**Figure 5B**), IL-6 (**Figure 5C**) and TNF-α (**Figure 5D**) at later stage (12-24 h post-stimulation of *M.tb*-EST12). Similarly, we found that EST12 induced iNOS (**Figure 5E**), NO (**Figure 5F**), IL-6 (**Figure 5G**) and TNF-α (**Figure 5H**) expression dependent on JNK, and JNK inhibitor significantly arrested EST12-induced effects (**Figures 5E–H**). Altogether, above results demonstrated that *M.tb*-EST12 decreased M2 polarization-related factor Arg1 or Myc expression and increased pro-inflammatory response and classical M1 activation dependent on RACK1 and JNK at later stage.

### Host E3 ubiquitin ligase FBW7 mediates the K48 ubiquitination and degradation of Myc at late stage

**Figure 6:**
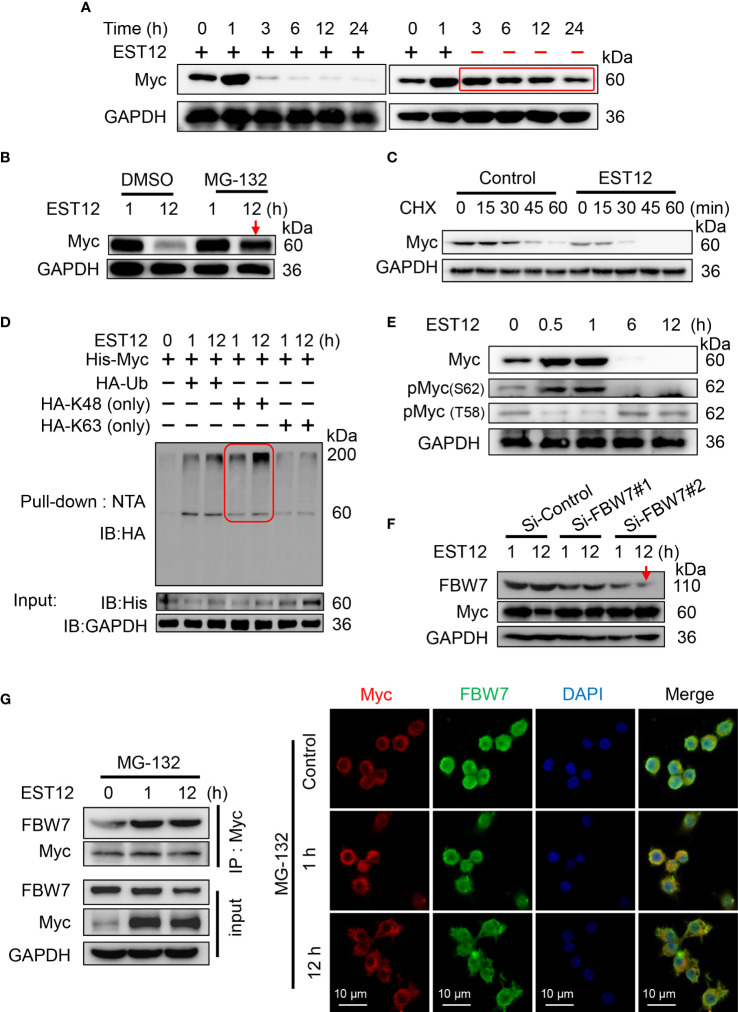
E3 ubiquitin ligase FBW7 mediates Myc K48 ubiquitination and degradation. **(A)** EST12 protein (2 μM) was added to stimulate RAW264.7 cells for 1 h. Then supernatants were replaced with fresh media to remove EST12, and cells were further incubated for the indicated time. The cell lysates were collected and Myc expression was analyzed by Western blot. **(B)** RAW264.7 cells were pretreated with MG-132 (5 μM), and then stimulated with EST12 protein (2 μM) for 1 or 12 h. Then the expression of Myc was analyzed by Western blot. **(C)** RAW264.7 cells were stimulated with EST12 protein (2 μM) for 1 h, then treated with CHX (20 μg/ml) for the indicated time, the expression of Myc was detected by Western blot. **(D)** RAW264.7 cells were transfected with the indicated plasmids, and then cell lysates were pulled down using Ni-NTA agarose. The ubiquitylation level of Myc was determined by Western blot with antibody against HA tag. **(E)** RAW264.7 cells were stimulated with EST12 protein (2 μM) for indicated time, the expression of Myc, pMyc (S62) and pMyc (T58) were detected by Western blot. **(F)** After RAW 264.7 cells were transfected with indicated siRNA for 36 h, cells were treated with EST12 protein (2 μM) for 1 or 12 h, then the expression of Myc and FBW7 were analyzed by Western blot. **(G)** MG-132-pretreated RAW264.7 cells were stimulated with EST12 protein (2 μM) for the indicated time, co-immunoprecipitation was performed with antibody against Myc. The expression of Myc and FBW7 was detected by Western blot (left), and the expression and co-localization between Myc (red) and FBW7 (green) were determined by confocal fluorescence microscopy (right). Scale bar: 10 μm.

As shown in **Figure 1E**, we found that EST12 stimulation induced Myc protein expression in macrophages at early stage (within 1h stimulation of EST12) and then decreased after 1 h. In order to detect whether late degradation of Myc protein is specific to EST12, we removed EST12 protein by replacing fresh media after 1 h stimulation of RAW264.7 cells with EST12 as indicated in **Figure 6A**, and found that Myc protein late degradation was significantly alleviated (**Figure 6A**). This result suggests that EST12 specifically induces early expression and late degradation of Myc protein. We further found that ubiquitin proteasome inhibitor MG-132 blocked EST12-induced Myc degradation (**Figure 6B**). Cycloheximide (CHX, a protein synthesis inhibitor) chase assay further demonstrated that Myc displayed significantly shortened half-life in EST12-treated RAW264.7 cells compared to the control group (**Figure 6C**). Furthermore, co-transfection experiment demonstrated that EST12 specifically upregulated K48 ubiquitination of Myc but not K63 ubiquitination (**Figure 6D**). Further, we found that after EST12 stimulation, dephosphorylation at Ser62-Myc and phosphorylation at Thr58-Myc at later stage (after 6 h stimulation of EST12, **Figure 6E**) was observed, suggesting that EST12 mainly triggers Myc Thr58 phosphorylation, but not Ser62, during the K48 ubiquitination degradation process of Myc at later stage (**Figure 6E**). Previous reports have shown that pS62-Myc is efficiently dephosphorylated by protein phosphatase 2A (PP2A) and T58-Myc is ubiquitinated by the E3 ubiquitin ligase SCF-FBW7, thus ultimately leading to proteosome-mediated degradation of Myc (18). Therefore, what we observed (in **Figure 6E**) might be the results of dephosphorylation at Ser62-Myc and phosphorylation at Thr58-Myc at later stage, which facilitates the ubiquitination degradation process of Myc.

To further identify which E3 ubiquitin ligase mediated EST12-induced Myc degradation, we silenced the best-studied Myc specific E3 ubiquitin ligase FBW7 with siRNAs. As shown in **Figure 6F**, Si-FBW7#2 siRNA-mediated FBW7 knockdown significantly hindered EST12-induced Myc degradation. Furthermore, MG-132-pretreated RAW264.7 cells were stimulated with EST12 for 1 or 12 h, co-immunoprecipitation (**Figure 6G**, left) and immunofluorescence (**Figure 6G**, right) experiments were performed to explore the interaction between Myc and E3 ubiquitin ligase FBW7. As shown in **Figure 6G**, although EST12 does not induce the expression of FBW7, it significantly increased the binding of FBW7 to Myc (**Figure 6G**, left), and EST12 treatment for 1h and 12 h promoted the colocalization of FBW7 and Myc as indicated by immunofluorescence assay (**Figure 6G**, red and green merged as yellow part). A recent work reported that eleven–nineteen lysine-rich leukemia (ELL) could also function as an E3 ubiquitin ligase, target Myc proteasomal degradation and suppress tumor growth (19). However, we did not found ELL was involved in EST12-induced K48 ubiquitination and degradation of Myc, since knockdown of ELL expression did not increase Myc expression (**Figure S3D**).

Taken together, these results demonstrated that host E3 ubiquitin ligase FBW7 bound with Myc and mediated EST12-induced Myc ubiquitination and degradation.

### EST12 induces Myc expression and activation through endocytosis of macrophage not through binding to TLR2/4 of macrophage

**Figure 7:**
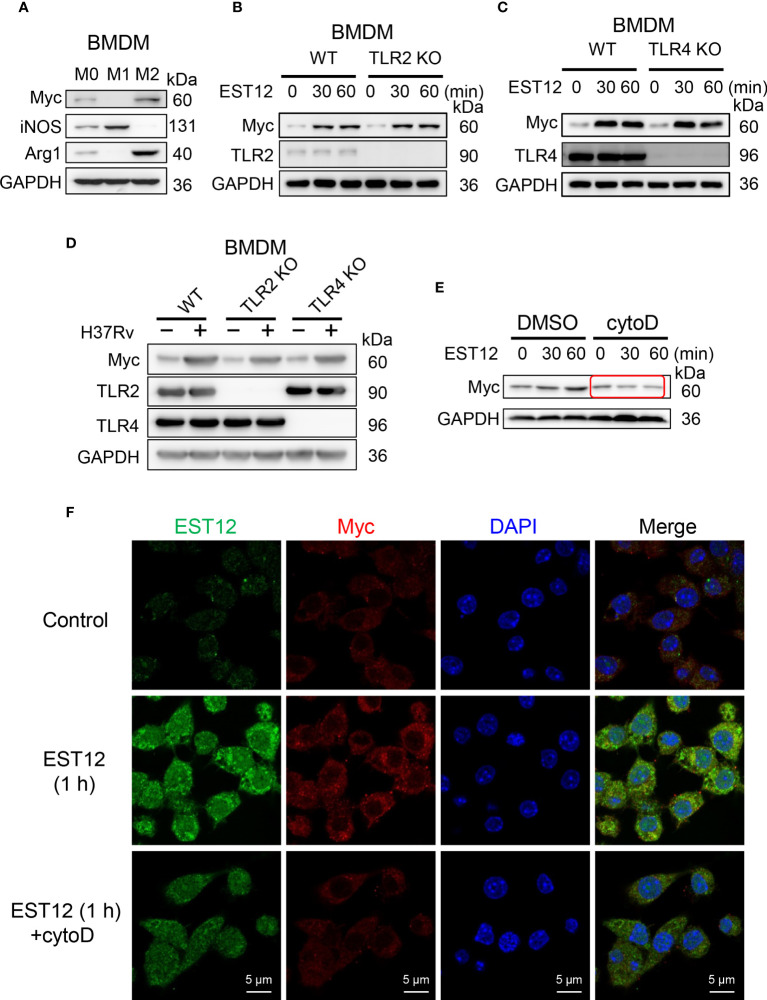
EST12 induces Myc expression and activation through endocytosis but not through binding of TLR2/4 of macrophage. **(A)** Myc expression was analyzed in M0-, M1- and M2-BMDMs by Western blot. **(B, C)** BMDMs isolated from WT, TLR2 KO **(B)** or TLR4 KO **(C)** mice were stimulated with EST12 protein (2 μM) for the indicated time, the expression of Myc was analyzed by Western blot. **(D)** BMDMs isolated from WT, TLR2 KO and TLR4 KO C57BL/6 mice, respectively, were infected with H37Rv at MOI = 10 for 24 h, the expression of Myc was detected by Western blot. **(E, F)** RAW264.7cells were stimulated with EST12 protein (2 μM) for 1 h in the presence or absence of Cytochalasin D (2.5 μM), the expression of Myc was detected by Western blot **(E)** and immunofluorescence **(F)**.

To explore whether EST12 upregulates Myc expression of macrophage through binding to TLR2/4 receptors of macrophages, BMDMs were used. Consistently with the previous report that Myc is expressed in BMDMs M0 (20), we found that Myc could be expressed in M2 and weakly expressed in M0 BMDMs, but not in M1 BMDMs (**Figure 7A**). Both WT M0 BMDMs and TLR2/4 KO M0 BMDMs were stimulated with EST12 protein (**Figures 7B, C**) or infected with *M.tb* H37Rv (**Figure 7D**). As evidenced in **Figures 7B–D**, TLR2 or TLR4 deficiencies did not decrease EST12 or *M.tb* H37Rv-induced Myc expression. However, when we pretreated RAW264.7 cells with 2.5 μM phagocytosis inhibitor cytochalasin D, we found that EST12-induced Myc expression was significantly blocked (**Figure 7E**). Consistently, as shown in immunofluorescence assay, cytochalasin D significantly blocked macrophage uptake of EST12 protein and macrophage Myc expression (**Figure 7F**), suggesting mycobacterial secreted EST12 protein enters cells through endocytosis not through binding to TLR2/4 of macrophages.

## Discussion

**Figure 8:**
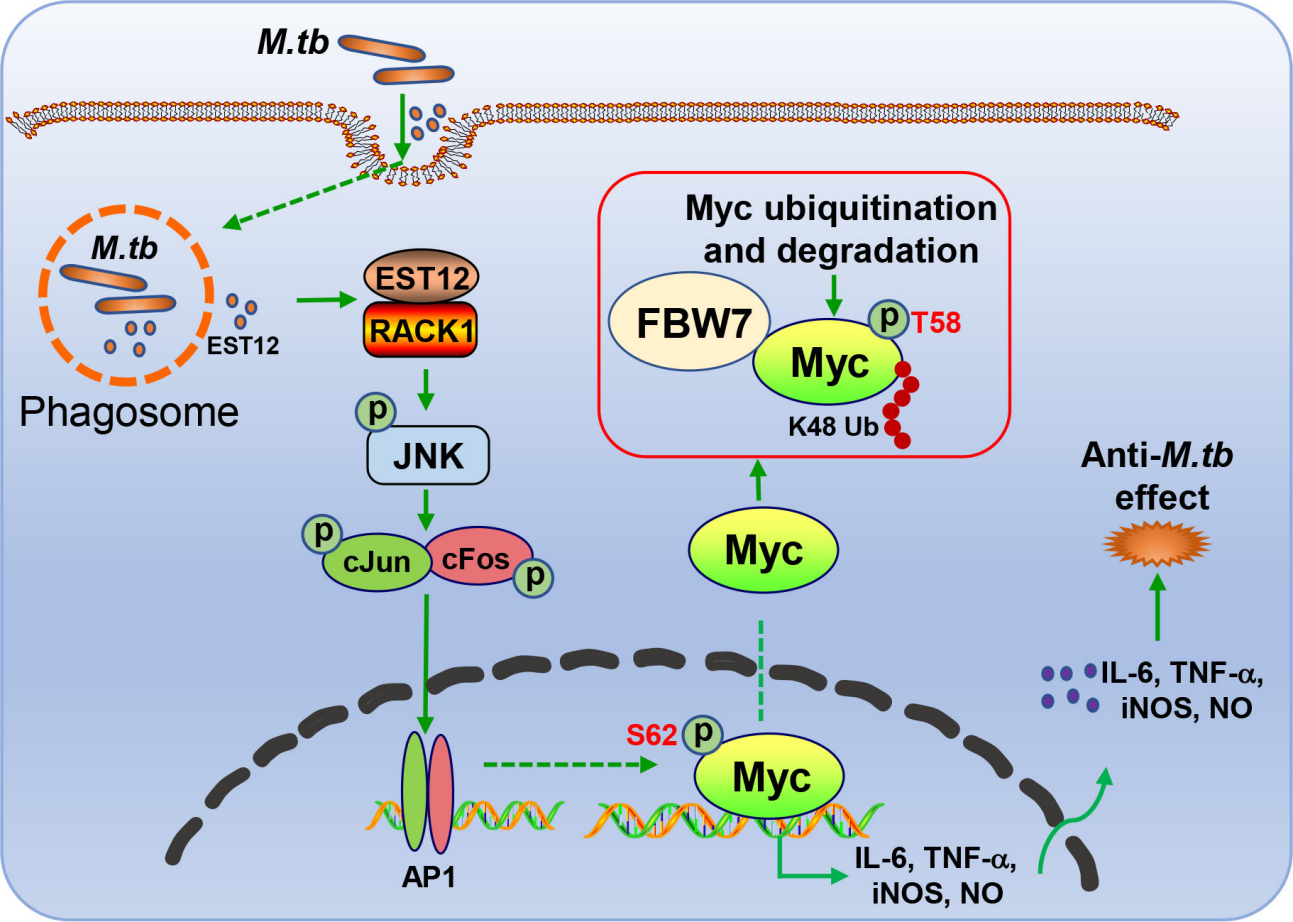
Schematic diagram of EST12-induced anti-*M.tb* immune effect through RACK1-JNK-AP1-Myc signaling axis.

In this study, we found that *M.tb*-EST12 protein and *M.tb* H37Rv infection induced the expression of Myc in both human THP1 cells and mouse macrophages (RAW264.7 and BMDMs). Importantly, we demonstrated that EST12 protein induced early expression and late K48 ubiquitination and degradation of Myc protein. EST12 activates JNK-AP1-Myc signaling pathway, promotes Myc to bind to the promoters of IL-6, TNF-α and iNOS, and then induces the expression of IL-6, TNF-α and iNOS, which further contributes to anti-mycobacterial effects. Our data suggest that Myc acts as a critical transcriptional factor and plays a pivotal role in *M.tb*-induced innate immunity (**Figure 8**).

Macrophages are the main effective immune cells in anti-TB immune response through mediating the pro-inflammatory response and immune clearance of *M.tb*. It is well known that classically activated M1 macrophages are the most effective immune cells for killing *M.tb* (21). JNK pathway has been reported to contribute to the expression of various pro-inflammatory cytokines such as IL-6 for *M.tb* clearance (22). The expression levels of various cytokines and chemokines play important roles in *M.tb* infection (23). Among them, *M.tb* infection promotes the expression of iNOS, or secretion of NO, IL-1β, TNF-α and IL-6 (24, 25). IL-1β directly kills *M.tb* or upregulates TNF-α expression and promotes macrophages apoptosis to eliminate *M.tb* (26, 27). Both IL-6 and TNF-α could increase the elimination of intracellular *M.tb* (10). IL-6 is a pleotropic cytokine that regulates both pro- and anti-inflammatory cytokine production (28). IL-6 knockdown resulted in increased bacterial burden in *M.tb* infected mouse macrophages and IL-6-deficient mice are susceptible to *M.tb* infection, indicating that IL-6 has an effect on the protective immune response against *M.tb* (29, 30). Other report also showed that mycobacteria- induced Myc expression is associated with the induction of inflammatory cytokines IL-6 and TNF-α and with the suppression of intracellular mycobacterial growth (10). Consistently, here we found that mycobacterial EST12 protein induced IL-6 and TNF-α expression of macrophages associated with anti-*M.tb* function (**Figures 3M, N**). Both IL-6 and TNF-α acted as pro-inflammatory cytokines and exerted the anti-*M.tb* function, since IL-6/TNF-α deficiency increased the survival of intracellular mycobacteria (**Figures 3M, N**). Recently, we have demonstrated that *M.tb*-EST12 could induce ROS expression (12), and ROS contributed to iNOS expression (18). Here, we demonstrated *M.tb* EST12 increased iNOS, NO, IL-6 and TNF-α expression and increased *M.tb* clearance depend on RACK1, Myc and JNK in macrophages (**Figures 3I–L**), and IL-6 KO or anti-TNF-α antibody treatment abrogated these effects. Since Myc is essential for cell survival and knockout of Myc is lethal to mice (data not shown). Unfortunately, we were unable to obtain macrophage conditional Myc KO homozygous mice to perform *in vivo* experiment.

Myc has been reported to be critical for the induction and maintenance of the M2-like macrophage state (31). Our results in **Figure 3A** suggest that EST12-induced Myc expression in a trend consistent with that of Arg-1 expression with M2-like macrophage phenotype. It was also reported that silencing of Myc attenuated BCG-induced IL-6 and TNF-α expression (10). However, the mechanism of Myc-induced IL-6 and TNF-α has not been reported yet. In this report, we firstly clarify how Myc induces the expression of IL-6 and TNF-α, and determine that Myc acts as a critical transcription factor to bind the promoters of these genes and induce their expression. We demonstrated Myc inhibitor decreased EST12 or H37Rv-induced IL-6 and TNF-α expression.

As a transcription factor, Myc could rapidly degrade following synthesis, and its half-life was about 20 min (32). Ubiquitin–proteasome system (UPS) is the most important mechanism for regulating Myc protein level (33). And FBW7 is the best-studied E3 ubiquitin ligase for Myc (8). Consistently, we found that FBW7 interacted with Myc and mediated EST12-induced K48 ubiquitination and degradation of Myc at late stage (**Figures 6D, F, G**).

Previous reports showed that the increased phosphorylation of Myc S62 promoted the stability of Myc protein, and phosphorylated S62 would also help Myc bind to GSK3β, which mediated phosphorylation of T58 and Myc ubiquitination degradation by FBW7 (32). In the present study, we found that EST12 could activate Myc by promoting S62 phosphorylation, decreasing T58 phosphorylation at the early stage (within 1 h stimulation of EST12) (**Figure 2A**). EST12 stimulation induced T58 phosphorylation of Myc at later stage (6 h post-stimulation of EST12) (**Figure 6E**), which might ultimately lead Myc to bind and recruit FBW7 for K48 poly-ubiquitination and degradation of Myc. A latest research report proposed a new mechanism for Myc S67 phosphorylation to antagonize GSK3β binding and thereby enhance the stability of Myc protein (34). Since antibody against Myc S67 phosphorylation is not commercially available, whether EST12 could induce Myc S67 phosphorylation requires further investigation in future.

Our results suggest that EST12-induced early Myc expression is beneficial for the induction of pro-inflammatory cytokines (such as IL-6 and TNF-α), which may contribute to host defense against mycobacteria. On the other hand, EST12 induces late K48 ubiquitination and degradation of Myc protein and decreases M2 polarization related factor Arg1 or Myc expression and increases classical M1 activation (iNOS/NO expression) at later stage (after 12 hour stimulation of EST12), which may also contribute to enhancement of anti-mycobacterial immunity. Previous reports have shown that c-Myc plays a positive role in phagocytosis, contributing to host defense against mycobacteria (35). Because of Myc-targeted gene network (9), it is highly possible for Myc to directly or indirectly affect phagocytosis or other processes, which need to be further investigated. As other signaling pathways (such as NF-κB mediated pathway) were also reported to mediate *M.tb*-induced inflammatory response (36), Myc might not be the only trigger of this process.

In addition, it has recently been reported that TB aggravates the progress of COVID-19 infection which accompanied cytokine storm with elevated IL-6 and TNF-α (37). Here both *M.tb* and *M.tb*-EST12 significantly induced pro-inflammatory cytokines IL-6 and TNF-α expression, suggesting that *M.tb*-induced pro-inflammatory cytokines might possibly have implication on inflammation of severity COVID-19.

Taken together, our findings suggest that *M.tb* secreted effector protein EST12 regulates early expression and late K48 ubiquitination and degradation of Myc protein, EST12 regulates Myc expression and enhances anti-mycobacterial inflammatory response *via* RACK1-JNK-AP1-Myc immune pathway. Our finding provides new insights into *M.tb*-induced immunity through Myc.

## Data availability statement

The raw data of sequencing has been deposited at GEO database. Accession numbers: RNA-seq (GSE210088), Cut&Tag-seq (GSE210089).

## Ethics statement

The animal study was reviewed and approved by committee on ethics in the Care and Use of Laboratory Animals of Wuhan University.

## Author contributions

X-LZ designed and supervised the research. JW conducted the experiments. HX, GL, and YG gave helps to the experiments. JW, X-LZ, F-LL, and YX analyzed the data and wrote the manuscript. All authors contributed to the article and approved the submitted version.

## Funding

This study was supported by grants from the National Grand Program on Key Infectious Disease of China (2017ZX10201301-006 and 2012ZX10003002-015), the National Key R&D Program of China (2018YFA0507603), the National Natural Science Foundation of China (91740120 and 22077097), the National Outstanding Youth Foundation of China (81025008), Hubei Province’s Outstanding Medical Academic Leader Program (523-276003), Hubei Province Key R&D Program (2020BCB020), Research and Innovation Team Project of Hubei Provincial Health Commission (WJ2021C002), the Medical Science Advancement Program (Basic Medical Sciences) of Wuhan University (TFJC 2018002), the Fundamental Research Funds for the Central Universities and Foundation Committee Innovation Group Project (21721005).

## Acknowledgments

The author would like to thank professor Jiahuai Han of Xiamen University for kindly providing the template of Myc DNA.

## Conflict of interest

The authors declare that the research was conducted in the absence of any commercial or financial relationships that could be construed as a potential conflict of interest.

## Publisher’s note

All claims expressed in this article are solely those of the authors and do not necessarily represent those of their affiliated organizations, or those of the publisher, the editors and the reviewers. Any product that may be evaluated in this article, or claim that may be made by its manufacturer, is not guaranteed or endorsed by the publisher.
