# Supplementary material for: EST12 regulates Myc expression and enhances anti-mycobacterial inflammatory response via RACK1-JNK-AP1-Myc immune pathway
Source: Front Immunol. 2022 Aug 8;13:943174. doi: 10.3389/fimmu.2022.943174 (PMC9393728; doi:10.3389/fimmu.2022.943174)
Supplement: Supplementary file 1 [file DataSheet_1.docx]

Supplementary Materials

Supplementary Figures

Supplementary Figure 1


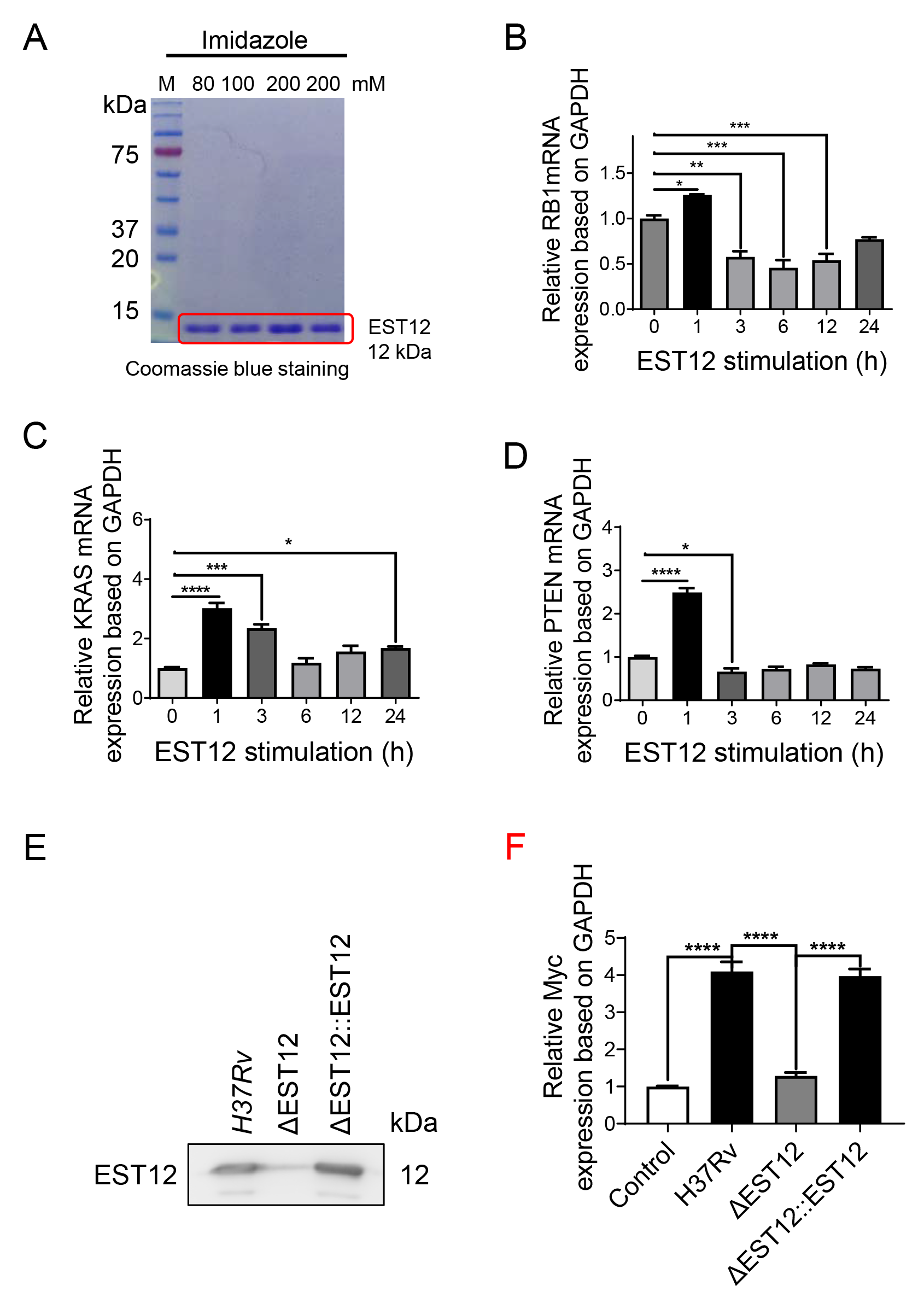


**Figure S1. Verification of EST12 effects on RB1/KRAS/PTEN mRNA expression, and EST12 expression in H37RV, ΔEST12 and ΔEST12::EST12. (A)** Coomassie brilliant blue staining of the purified recombinant EST12 protein. **(B-D)** Effect of EST12 stimulation on RB1, KRAS and PTEN mRNA expression in RAW264.7 cells. RAW264.7 cells were stimulated with EST12 protein (2 μM) for the indicated time, the mRNA levels of RB1, KRAS and PTEN were detected by RT-qPCR. **(E)** Confirmation of the expression of EST12 in the indicated strains by Western blot with EST12 polyclonal rabbit antibody. **(F)** Western blot bands in **Figure 1F** were quantified using ImageJ. One-way ANOVA with Dunnett’s multiple comparisons test was used to assess the statistical significance in **B**-**D** and **F.** The data are expressed as the mean ± SEM of three independent experiments, *p* > 0.05, not significant (ns); **P* < 0.05, ***P* < 0.01, ****P* < 0.001, or *****P* < 0.0001.

Supplementary Figure 2


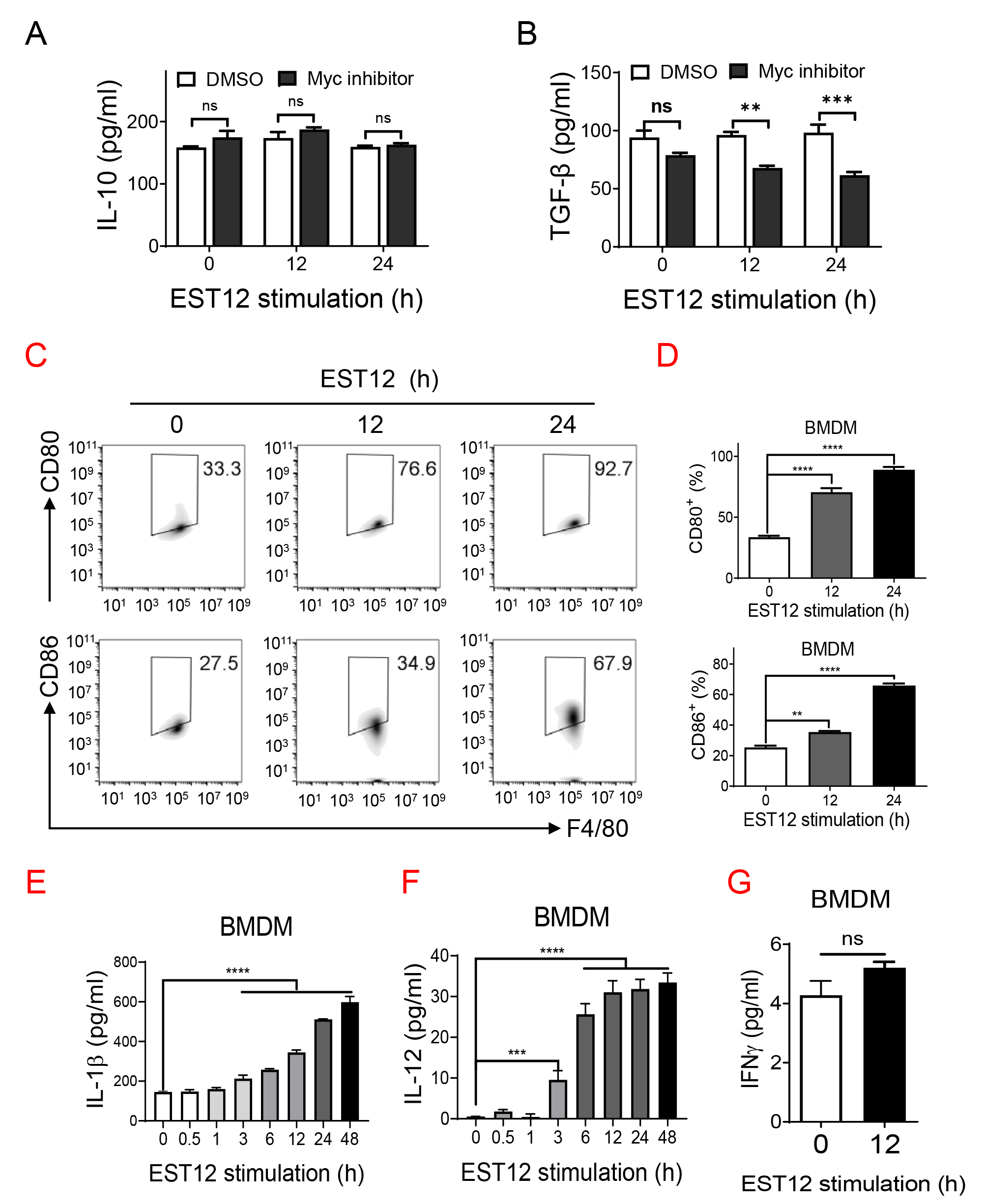


Figure S2. Analysis of the effects of EST12 on the expression of IL-10, TGF-β, CD80, CD86, IL-1β, IL-12 and IFN-γ. (A, B) Myc inhibitor (20 μM)-pretreated RAW264.7 cells were stimulated with EST12 protein for the indicated time, the levels of the secreted IL-10 (A) and TGF-β (B) were determined by ELISA. (C-G) BMDMs were stimulated with EST12 protein (2 μM) for the indicated time, the expression of CD80 and CD86 was analyzed by FCM (C, D) and the concentrations of IL-1β (E), IL-12 (F) and IFNγ (G) in the supernatants were analyzed by ELISA. C. The representative FCM analysis of CD80/CD86 expression in EST12-stimulated BMDMs from C57BL/6 mice. D. The statistical analysis for the data of FCM in C. Two-way ANOVA with Tukey’s multiple comparisons test in A-B, and One-way ANOVA with Tukey’s multiple comparisons test in D-F were used to assess the statistical significance. Two-tailed unpaired Student’s t test was used to assess the statistical significance in G (*vs* 0 h). The data are expressed as the mean ± SEM of three independent experiments, *p* > 0.05, not significant (ns), ***P* < 0.01, ****P* < 0.001.

Supplementary Figure 3


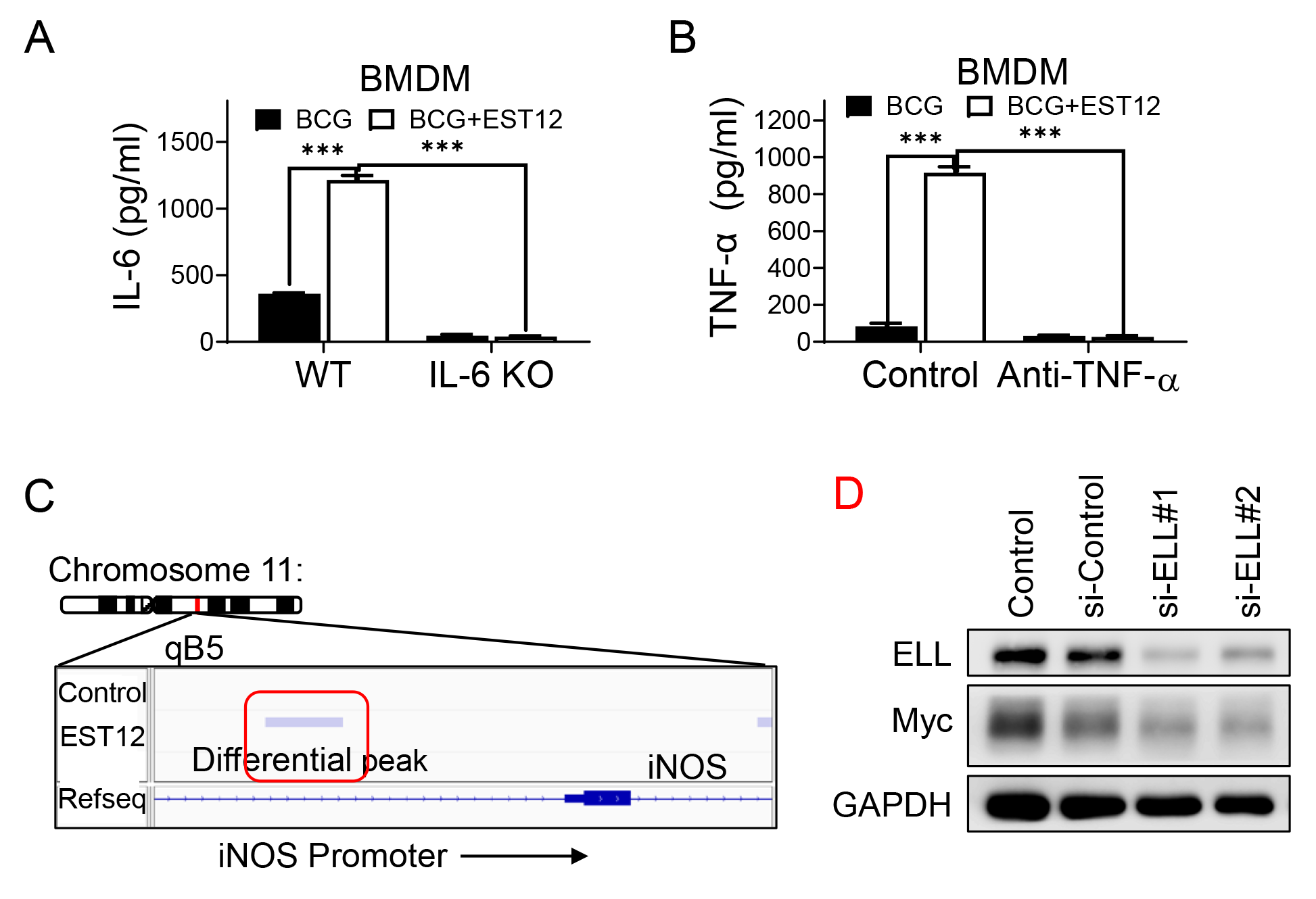


**Figure S3.Analysis of the effects of EST12 on the expression ofIL-6/TNF-α/iNOS.**  **(A, B)** BMDMs (5 ×10^5^) isolated from WT or IL-6 KO mice were infected with BCG at MOI=10 for 4 h. Then cells were washed with PBS for three times and subsequently stimulated with EST12 (2 μM) for 8 h in the presence of TNF-α neutralizing antibody (10 μg/ml) or not. The secreted IL-6 **(A)** or TNF-α **(B)** in the supernatants were determined by ELISA. **(C)** RAW264.7 cells were stimulated with EST12 protein (2 μM) for 1 h, and then proceeded for CUT&Tag sequencing analysis as described in Materials and Methods. **(D)** RAW264.7 cells were transfected with indicated siRNAs and the expression of ELL and Myc was determined by Western blot. Two-way ANOVA with Tukey’s multiple comparisons test was used to assess the statistical significance in **A, B**. The data are expressed as the mean ± SEM of three independent experiments. *p* > 0.05, not significant (ns), ****p* < 0.001.

**Supplementary Table 1. Primers used in RT-qPCR**

| **Primer name** | **Sequence** | **Melting Temperature (**°C**)** | **Product Length (bp)** |
| --- | --- | --- | --- |
| IL-6 F | GTCCTTCCTACCCCAATTTCCA | 59.7 | 154 |
| IL-6 R | TAACGCACTAGGTTTGCCGA | 59.7 | 154 |
| TNF-α F | GATCGGTCCCCAAAGGGATG | 60 | 131 |
| TNF-α R | GGTTTGCTACGACGTGGGC | 61.7 | 131 |
| Myc F | 5’CGTTGGAAACCCCGCAG 3’ | 60.1 | 86 |
| Myc R | 5’TACGGAGTCGTAGTCGAGGT 3’ | 54.6 | 86 |
| Rb1 F | 5’TGGAAGCCAACTTGACAAGAGA3’ | 59.9 | 164 |
| RB1 R | 5’GCTGAGAGGACAAGCAGGTT3’ | 56.7 | 164 |
| KRAS F | 5’GAGCGCCTTGACGATACAG3’ | 56.4 | 81 |
| KRAS R | 5’TGTTTCCTGTAGGAGTCCTC3’ | 51.2 | 81 |
| PTEN F | 5’TTCAAGTAAAATTTTCCCACTG 3’ | 54.7 | 186 |
| PTEN R | 5’TGGTAGCCAAACGGAACTTCA 3’ | 60.7 | 186 |
| GAPDH F | 5’TGTTTCCTCGTCCCGTAG 3’ | 53.6 | 108 |
| GAPDH R | 5’CAATCTCCACTTTGCCACT 3’ | 52.8 | 108 |

**Supplementary Table 2. siRNA sequences for FBW7 silencing**

| **siRNA name** | **Sequence** |
| --- | --- |
| siRNA-FBW7#1 | 5’-UCAGAUUCAAGUUGGUUAATT-3’ |
| siRNA-FBW7#2 | 5’-GGUUUGAUUCACUUAGAAATT-3’ |
| siRNA-control | 5’-UUCUCCGAACGUGUCACGCTT-3’ |

**Supplementary Table 3. Primers used in ChIP-qPCR**

| **Primer name** | **Sequence** |
| --- | --- |
| IL-6#1 F | 5’AATCCTAGCCTCTTATTCA3’ |
| IL-6#1 R | 5’CACAATGTTTTATAAGGAG3’ |
| IL-6#2 F | 5’CTCCTTATAAAACATTGTG3’ |
| IL-6#2 R | 5’TAGCCCTAAGAAGCATGAG3’ |
| TNF-α#1 F | 5’TTCCACGTCGCGGATCATG 3’ |
| TNF-α#1 R | 5’GAACATCTTGGAAATAGCT3’ |
| TNF-α#2 F | 5’AGCTATTTCCAAGATGTTC3’ |
| TNF-α#2 R | 5’CTTTTCCCCGCCCTCTTCC3’ |

**Supplementary Table 4. Probes used in EMSA assay**

| **Probe name** | **Probe sequence** |
| --- | --- |
| probe for IL-6 | 5’AATCCTAGCCTCTTATTCATGTGTGTGTGTG-3’ |
| Mutant probe for IL-6 | 5’AATCCTAGCCTCTTATTGCACGATGTGTGTG-3’ |
| probe for TNF-α | 5’CTGCTGGCTGGCTGTGCAGACGGCCGCCTTT-3’ |
| Mutant probe for TNF-α | 5’CTGCTGGCTGGCTGTGATAGTAGCCGCCTTT-3’ |

The underlined regions are the mutated sequences relative to the wild type sequences.

**Supplementary Table 5. Primers used in plasmid construction**

| **Primer name** | **Sequence** |
| --- | --- |
| Myc F | 5’ ATGAATTCCAATGGCGGCGCTGAAGGAGGA 3’ |
| Myc R | 5’ TAGGATCCGGGCCAAGCCTGCAGCTGCCGCTGG 3’ |
| IL6-1 F | 5’ *TACGCGTGCTAGCCCGGG*ACCAAAGGGAAGAAGTCTGTTT 3’ |
| IL6-2 F | 5’ *TACGCGTGCTAGCCCGGG*AATAGGCTTGGACTTGGAAGCC 3’ |
| IL6-3 F | 5’ *TACGCGTGCTAGCCCGGG*CTTATAAAACATTGTGAATTTCAG 3’ |
| IL-6 R | 5’ *CAGTACCGGAATGCC*ACTTGCAGAGAGGAACTTCATAGCG 3’ |
| TNF-α-1 F | 5’ *TACGCGTGCTAGCCCGGG*TTTAAGAGTGAGCCTGGTATTGTG 3’ |
| TNF-α-2 F | 5’ *TACGCGTGCTAGCCCGGG*AGACCAGGCTGGCCTCGAACTCA 3’ |
| TNF-α-3 F | 5’ *TACGCGTGCTAGCCCGGG*TATGGGAGCACTGGCCTTGATA 3’ |
| TNF-α R | 5’ *CAGTACCGGAATGCC*CATTTACTGCTTTGGAGTCACAGAC 3’ |

The underlined regions are the restriction enzyme digestion sites for *Eco*RI and *Bam*HI. The italic regions are the homology arm for pGL3 vector.

**Additional data can be accessed at the following websites:**

1, Transcriptional sequencing, Figure 1A:<https://www.jianguoyun.com/p/DUw5c9EQu-XJChi17b8EIAA>

2, CUT&TAG results: <https://www.jianguoyun.com/p/DQ7EQRcQu-XJChiR7b8EIAA>

3, Statistical chart data: <https://www.jianguoyun.com/p/DfHxbRYQu-XJChi77b8EIAA>

4, Original images: <https://www.jianguoyun.com/p/DYeKN0IQu-XJChiC7b8EIAA>
